# The Antioxidant Procyanidin Reduces Reactive Oxygen Species Signaling in Macrophages and Ameliorates Experimental Colitis in Mice

**DOI:** 10.3389/fimmu.2017.01910

**Published:** 2018-01-05

**Authors:** Lu Chen, Qian You, Liang Hu, Jian Gao, Qianqian Meng, Wentao Liu, Xuefeng Wu, Qiang Xu

**Affiliations:** ^1^State Key Laboratory of Pharmaceutical Biotechnology, School of Life Sciences, Nanjing University, Nanjing, China; ^2^Department of Pharmacy, Sir Run Run Shaw Hospital Affiliated to Nanjing Medical University, Jiangsu, China; ^3^Department of Pharmacology, Jiangsu Key Laboratory of Neurodegeneration, Nanjing Medical University, Jiangsu, China

**Keywords:** procyanidin, inflammatory bowel disease, reactive oxygen species signaling, MMP9, NF-κB, NLRP3 inflammasome

## Abstract

Management of inflammatory bowel disease (IBD) is a real clinical challenge. Despite intense investigation, the mechanisms of IBD remain substantially unidentified. Some inflammatory conditions, such as matrix metalloproteinases (MMPs) and the nuclear factor-κB (NF-κB) and NOD-like receptor protein 3 (NLRP3) inflammasome signaling pathways, are reported to contribute to the development and maintenance of IBD. Regulation of their common upstream signaling, that is, reactive oxygen species (ROS), may be important to control the progression of IBD. In the present study, we found that procyanidin, a powerful antioxidation flavonoid, has a significant effect on ROS clearance on THP-1 macrophages after lipopolysaccharide (LPS) or LPS-combined adenosine triphosphate stimulation, thus downregulating MMP9 expression, suppressing NF-κB signaling, and interrupting the formation of the NLRP3 inflammasome. Moreover, our *in vivo* data showed that procyanidin attenuated Dextran sulfate sodium-induced experimental colitis in a dose-dependent fashion by suppressing the expression of MMP9, NF-κB, and NLRP3 inflammasome signaling in colonic tissues in mice. Overall, our results suggested that targeting ROS could be a potential therapeutic choice for colonic inflammation.

## Introduction

Inflammatory bowel disease (IBD), including ulcerative colitis and Crohn’s disease, is characterized by chronic, relapsing inflammation that significantly lowers the quality of life of patients and even increases the risk of colon cancer (1, 2). However, the exact mechanism of IBD still remains unclear. It is widely accepted that disruption of the epithelial barrier triggers invasion of bacterial antigens into the mucosal layer, resulting in activation of the mucosal immune response (3). During this process, macrophages play a key role because they can release large amounts of proinflammatory cytokines, such as interleukin (IL)-1, IL-6, and tumor necrosis factor (TNF)-α, which exacerbate the severity of the inflammation condition and colitis (4).

Reactive oxygen species (ROS) are a class of highly reactive and unstable molecules that include hydrogen peroxide (H_2_O_2_), hydroxyl radical (OH^⋅^), singlet oxygen ${(}^{1}{\text{O}}_{2})$, and superoxide $\left({\text{O}}_{2}^{\cdot -}\right)$ (5). ROS are generated from mitochondria during adenosine triphosphate (ATP) synthesis, and excessive amounts of ROS can trigger a large amount of damage to DNA and protein. There is substantial evidence that ROS have a strong correlation with colitis (6) and are over-produced in the colon of IBD patients (7), suggesting a high level of oxidative stress during the development of colitis. In particular, excessive ROS, mainly produced by activated macrophages and leukocytes in the colon, induce a series of responses that prolong inflammation, such as matrix metalloproteinase (MMPs), toll-like 4 receptor (TLR4)-NF-κB, and NLRP3 signaling pathway activation, which play crucial roles in colitis.

Given the essential role of ROS in colitis, removal of ROS should be a therapeutic choice. Procyanidin is a type of flavonoid that is mainly found in grape skin, grape seeds, and green tea. Procyanidin has been demonstrated to have a powerful oxygen radical absorbance capacity, much stronger than those of vitamin C and vitamin E (8). In addition, previous studies have reported that procyanidin can alleviate many inflammatory conditions (9, 10). In the present study, we examined the anti-inflammatory effect of procyanidin in dextran sulfate sodium (DSS)-induced colitis and found that procyanidin decreased the generation of ROS, inhibiting the expression of MMP9, NF-κB, and formation of the NLRP3 inflammasome. Therefore, targeting ROS may be a potential strategy for inflammatory disease therapy.

## Materials and Methods

### Animals

6- to 8-week male C57BL/6 mice were obtained from the Model Animal Genetics Research Center of Nanjing University (Nanjing, China). Mice were grouped at SPF facility with controlled temperature (22 ± 2°C) and 12:12-h light–dark cycle. Animal welfare and experimental procedures were carried out strictly in accordance with the recommendation of Guide for the Care and Use of Laboratory Animals [Ministry of Science and Technology of China (11)], and the Nanjing University Animal Care and Use Committee (NJU-ACUC). The protocol was approved by the Nanjing University Animal Care and Use Committee (NJU-ACUC) and to minimize suffering and to reduce the number of mice used.

### Reagents

Procyanidin was purchased from Aladdin Co. Ltd. (Shanghai, China). Phorbolmyristate acetate (PMA), lipopolysaccharide (LPS), and ATP were purchased from Sigma-Aldrich (St. Louis, MO, USA). ATP was dissolved in ddH_2_O (500 mM) and 0.1 M NaOH was used to adjust the pH value to 7.4 as stock solution. DSS was bought from MP Biomedical (Aurora, OH, USA). RPMI-1640 and fetal bowel serum were purchased from Life Technology (Carlsbad, CA, USA). Antibody for F4/80 was purchased from eBioscience (San Diego, CA, USA). Antibodies for NLRP3, phospho-p65, total p65, phospho-IKKα/β, total IKKα, anti-total IKKβ, phospho-IκBα, total IκBα and caspase-1 were purchased from Cell Signaling Technology (Beverly, MA, USA). Antibody for apoptosis-associated speck-like protein containing a CARD (ASC) was purchased from Santa Cruz (Santa Cruz, CA, USA). Antibody for MMP9 was purchased from R&D System (Minneapolis, MN, USA). ELISA kits for human IL-1β were purchased from Dakewe Biotech Co. Ltd. (Beijing, China). All other chemicals were purchased from Sigma-Aldrich (St. Louis, MO, USA).

### Cell Culture

Human THP-1 cells were purchased from Shanghai Institute of Cell Biology (Shanghai, China) and maintained in RPMI 1640 medium, supplemented with 100 U/ml of penicillin, 100 µg/ml of streptomycin, and 10% fetal calf serum under a humidified 5% (v/v) CO_2_ atmosphere at 37°C.

### Measurement of Intracellular ROS

Cells (1 × 10^6^ per well) were cultured in a 6-well plate and treated with procyanidin in the presence or absence of LPS or ATP plus LPS for 6 h. Then, the cells were harvested and incubated with 2,7-dichlorofluorescein diacetate (DCFH-DA, Invitrogen, USA) at 37°C for 20 min and washed twice with cold PBS. DCF fluorescence distribution was detected by flow cytometry on a FACScan (Becton Dickinson, USA) at an excitation wavelength of 488 nm and an emission wavelength of 525 nm. Data were analyzed by Cell Quest software (Molecular Devices Corporation, CA, USA).

### Real-time Quantitative PCR

Real-time PCR was performed as described previously (12). Total RNA of colon tissue or THP-1 cells were reverse transcribed to cDNA and subjected to quantitative PCR, which was performed with the BioRad CFX96 Touch™ Real-Time PCR Detection System (BioRad, CA, USA) using iQTM SYBR^®^ GreenSupermix (BioRad, CA, USA), and threshold cycle numbers were obtained using BioRad CFX Manager software. The program for amplification was 1 cycle of 95°C for 2 min followed by 40 cycles of 95°C for 10 s, 60°C for 30 s, and 95°C for 10 s. β-actin was used as an endogenous control to normalize for differences in the amount of total RNA in each sample, and the relative gene expression was calculated using comparative C_T_ method also referred to as 2^−ΔΔCT^ method, i.e., fold change = 2^−ΔΔCT^ = [(C_T_ gene of interest − C_T_ gene of actin control) sample A − (C_T_ gene of interest − C_T_ gene of actin control) sample B]. The primer sequences used in this study were as follows:

Human-TNF-α Forward 5′-TGGCCCAGGCAGTCAGA-3′;
Human-TNF-α reverse 5′-GGTTTGCTACAACATGGGCTACA-3;
Human-IL-1β forward 5′-CTGATGGCCCTAAACAGATGAAG-3′;
Human-IL-1β reverse 5′-GGTCGGAGATTCGTAGCAGCTGGAT-3′;
Human-IL-6 forward 5′-GGTACATCCTCGACGGCATCT-3′;
Human-IL-6 reverse 5′-GTGCCTCTTTGCTGCTTTCAC-3′;
Human-β-actin forward 5′-CTCTCTGCTCCTCCTGTTCGAC-3′;
Human-β-actin reverse 5′-TGAGCGATGTGGCTCGGCT-3′.
Mouse-TNF-α forward 5′-CGAGTGACAAGCCTGTAGCCC-3′;
Mouse-TNF-α reverse 5′-GTCTTTGAGATCCATGCCGTTG-3;
Mouse-IL-1β forward 5′-CCAAGCTTCCTTGTGCAAGTA-3′;
Mouse-IL-1β reverse 5′-AAGCCCAAAGTCCATCAGTGG-3′;
Mouse-IL-6 forward 5′-CTGCAAGAGACTTCCATCCAGTT-3′;
Mouse-IL-6 reverse 5′-GAAGTAGGGAAGGCCGTGG-3′;
Mouse-β-actin forward 5′-TGCTGTCCCTGTATGCCTCT-3′;
Mouse-β-actin reverse 5′-TTTGATGTCACGCACGATTT-3′.
Mouse-MMP9 forward 5′-CTGGACAGCCAGACACTAAAG-3′
Mouse-MMP9 reverse 5′-CTCGCGGCAAGTCTTCAGAG-3′

### Western Blot

Samples were collected and lysed in a lysis buffer containing protease inhibitor (protease inhibitor cocktail, Pierce). The proteins were fractionated by SDS-PAGE and electrophoretically transferred onto polyvinylidene fluoride membranes. The membrane was blocked with 5% BSA for 2 h at room temperature. Different antibodies were incubated overnight at 4°C, and then incubated with secondary antibody. The software Quantity One (Bio-Rad Laboratories, Hercules, CA, USA) was used for densitometric analysis.

### Gelatin Zymography

THP-1 cells (1 × 10^6^ per well) were cultured in a 6-well plate and treated with 500 nM PMA to differentiate to macrophage for 3 h. Supernatant was collected after LPS (100 ng/ml) incubation in THP-1 cells in the absence or presence of procyanidin. After adding loading buffer to each sample, same volume was loaded into the wells of gels (8% polyacrylamide gels containing 0.1% gelatin). After electrophoresis, each gel was incubated with 50 ml of developing buffer for 48 h (37°C) in shaking bath. Then the gels were stained with coomassie brilliant blue (1%, with 10% acetic acid, 10% isopropyl alcohol, diluted with water).

### Induction of Colitis and Treatment

2.5% (wt/vol) DSS was used to induce acute colitis in mice. Mice were fed with DSS dissolved in drinking water continuously from day 0 to day 9, and then given normal drinking water for the next 2 days before sacrificed. Normal C57BL/6 mice received the same drinking water without DSS (*n* = 6–8 mice in each group). Procyanidin (10, 20, 40 mg/kg, i.g.) and mesalazine (200 mg/kg, i.g.) were dissolved in sodium salt of caboxy methyl cellulose (CMC-Na, 0.5%). Procyanidin and mesalazine were administered once a day from day 0 to day 11. The animals were observed once daily for weight, morbidity, and the presence of gross blood in feces and at the anus. The disease activity index (DAI) was calculated by assigning well-established and validated scores as previously described (13, 14). Briefly, the following parameters were used for calculation: (a) diarrhea (0 points = normal, 2 points = loose stools, 4 points = watery diarrhea); (b) hematochezia (0 points = no bleeding, 2 points = slight bleeding, 4 points = gross bleeding). At day 11 following induction with DSS, the animals were sacrificed, the entire colon was quickly removed for *ex vivo* study. Segments of the colon taken for histopathological essay were fixed in 10% normal buffered formalin, embedded in paraffin. Sections were stained with hematoxylin and eosin and histological score was evaluated (blinded) as follows: 0, no signs of inflammation; 1, low leukocyte infiltration; 2, moderate leukocyte infiltration; 3, high leukocyte infiltration, moderate fibrosis, high vascular density thickening of the colon wall, moderate goblet cell loss, and focal loss of crypts; and 4, transmural infiltrations, massive loss of goblet cell, extensive fibrosis, and diffuse loss of crypts.

### Immunofluorescence Histochemistry

F4/80 macrophage infiltration analysis was performed on paraffin-embedded colonic tissue sections (4–5 µm). Briefly, the sections were deparaffinized, rehydrated, and washed in 1% PBS-Tween20. Then, they were treated with 2% hydrogen peroxide, blocked with 3% BSA, and incubated for 2 h at room temperature with antibody for F4/80-FITC (1:100). The slides were then counter-stained with DAPI for 1 min. The reaction was stopped by thorough washing in PBS for 20 min. Images were acquired by confocal laser-scanning microscope (Olympus FV1000, Olympus, Japan). Settings for image acquisition were identical for control and experimental tissues.

### Immunohistochemical Analysis

Immunohistochemical analysis was performed on paraffin-embedded colon tissue sections (4–5 µm) to evaluate the p-p65 expression. Slides were deparaffinized, rehydrated, and blocked as described in Section “Gelatin Zymography,” and incubated with p-p65 antibody overnight at 4°C. Then, slides were incubated with streptavidin-HRP (Shanghai Gene Company, GK500705, Shanghai, China) for 40 min, then stained with DAB (Shanghai Gene Company, GK500705, Shanghai, China) substrate, and counter-stained with hematoxylin.

### ELISA Assay

The amounts of IL-1β in the cultured cells were quantified by ELISA kits according to the manufacturer’s instructions.

### Coimmunoprecipitation Assay

Proteins from cells (1 × 10^7^ per well) were incubated with 2 µg of appropriate antibody and precipitated with protein A/G-agarose beads (Santa Cruz Biotechnology, Santa Cruz, CA, USA). The immunoprecipitated proteins were separated by SDS-PAGE and western blot was performed with the indicated antibodies.

### Statistical Analysis

Results were expressed as mean ± SEM of three independent experiments and each experiment included triplicate sets. Data were statistically evaluated by one-way ANOVA followed by Dunnett’s test between control group and multiple dose groups. The level of significance was set at a *P*-value of 0.05.

## Results

### Procyanidin Inhibited the Generation of ROS in THP-1 Cells

**Figure 1:**
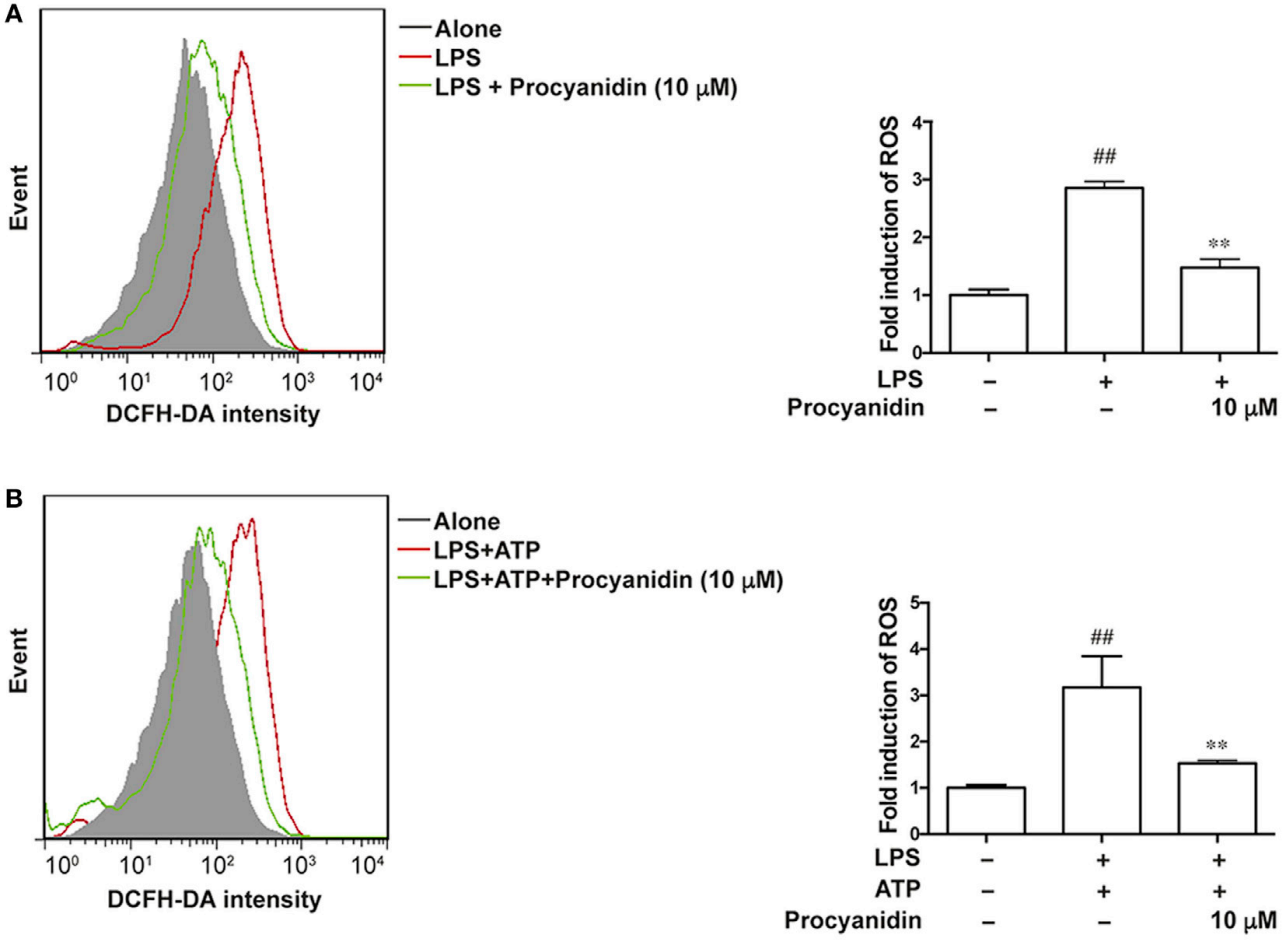
Procyanidin inhibited the generation of reactive oxygen species (ROS) in THP-1 cells. **(A)** THP-1 cells were pretreated with 500 nM phorbolmyristate acetate (PMA) for 3 h and were then cultured with 100 ng/ml lipopolysaccharide (LPS) for 2 h in the absence or presence of procyanidin (10 µM). **(B)** THP-1 cells were preincubated with 500 nM PMA for 3 h, followed by a 3 h treatment with procyanidin (10 µM) and 100 ng/ml LPS as well as a culturing with 5 mM adenosine triphosphate (ATP) for 1 h. ROS was stained with DCFH and measured by flow cytometry. ^##^*P* < 0.01 vs. vehicle group, **P < 0.01 vs. LPS alone or LPS + ATP-treated group.

Macrophages play a critical role in the initiation and progression of inflammation in IBD (15, 16), and ROS are predominantly generated from activated macrophages. We used THP-1 cells to examine the antioxidative effect of procyanidin. THP-1 cells were differentiated to macrophages by cultured with PMA (500 nM) for 3 h, and then single LPS (100 ng/ml) or LPS (100 ng/ml) combined with ATP (5 mM) stimulation—two classic methods of inducing activation of TLR4-NF-κB and the NLRP3 inflammasome, respectively—were applied to mimic the inflammatory condition in THP-1 cells. Under LPS challenge, ROS was generated by bind of TLR4 with NADPH oxidase 4 to produce superoxide (17) and according to former reference (14), ATP (5 mM) alone is also sufficient to induce ROS generation. In the present study, we found that the fold induction of ROS appeared to be the same with LPS and LPS + ATP, suggesting that the TLR pathway dominates the production of ROS. Since procyanidin has a powerful antioxidation effect, we investigated whether procyanidin also has an inhibitory effect on the production of ROS triggered by LPS or LPS plus ATP stimulation. As shown in Figures 1A,B, upon LPS stimulation or co-stimulation with LPS and ATP, increased amounts of ROS were generated in THP-1 cells, and procyanidin (10 µM) administration significantly suppressed the elevation of ROS.

### Procyanidin Downregulated Expression of MMP9 in THP-1 Cells

**Figure 2:**
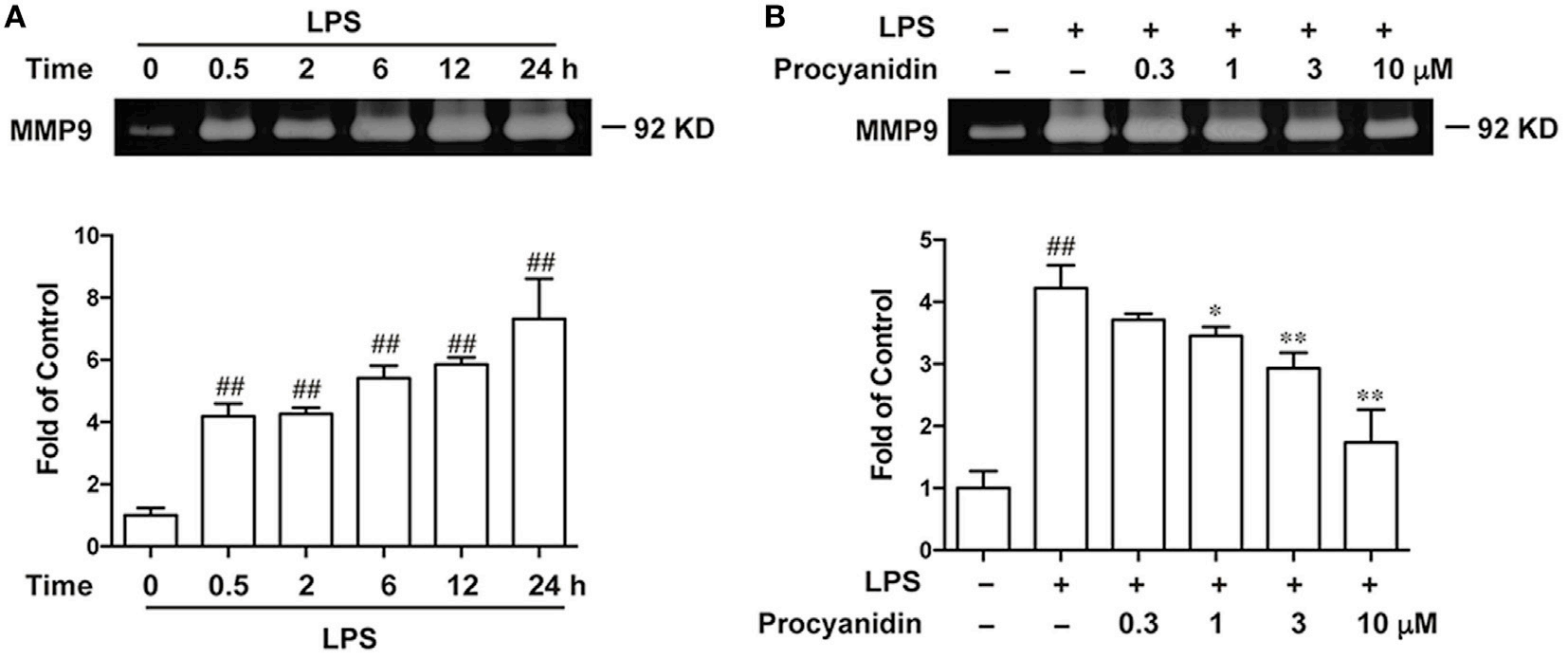
MMP9 expression was consistently upregulated following lipopolysaccharide (LPS) stimulation and was inhibited by procyanidin treatment in THP-1 cells. **(A)** THP-1 cells were preincubated with 500 nM phorbolmyristate acetate (PMA) for 3 h, followed by 100 ng/ml LPS for different times (0, 0.5, 2, 6, 12, and 24 h); supernatants were collected to measure MMP9 expression by gelatin zymography. **(B)** THP-1 cells were preincubated with 500 nM PMA for 3 h, followed by 24 h treatment with 100 ng/ml LPS in the presence or absence of procyanidin (0.3, 1, 3, and 10 µM); MMP9 expression was examined by gelatin zymography. ^##^*P* < 0.01 vs. vehicle group, *P < 0.05, **P < 0.01 vs. LPS alone.

Since LPS promotes the generation of ROS, which induce the activation of MMP9, we investigated whether procyanidin was able to inhibit MMP9. First, we examined the change in MMP9 expression by gelatin zymography during LPS stimulation for 0.5, 2, 6, 12, and 24 h. As shown in Figure 2A, elevated MMP9 was accompanied with LPS, especially in the 24th hour after LPS challenge. Next, we evaluated the inhibitory effect of procyanidin on MMP9 expression and found that procyanidin downregulated MMP9 from LPS administration for 24 h in a dose-dependent fashion (0.3, 1, 3, 10 µM) (Figure 2B).

### Procyanidin Decreased NLRP3 Inflammasome Activation in THP-1 Cells

**Figure 3:**
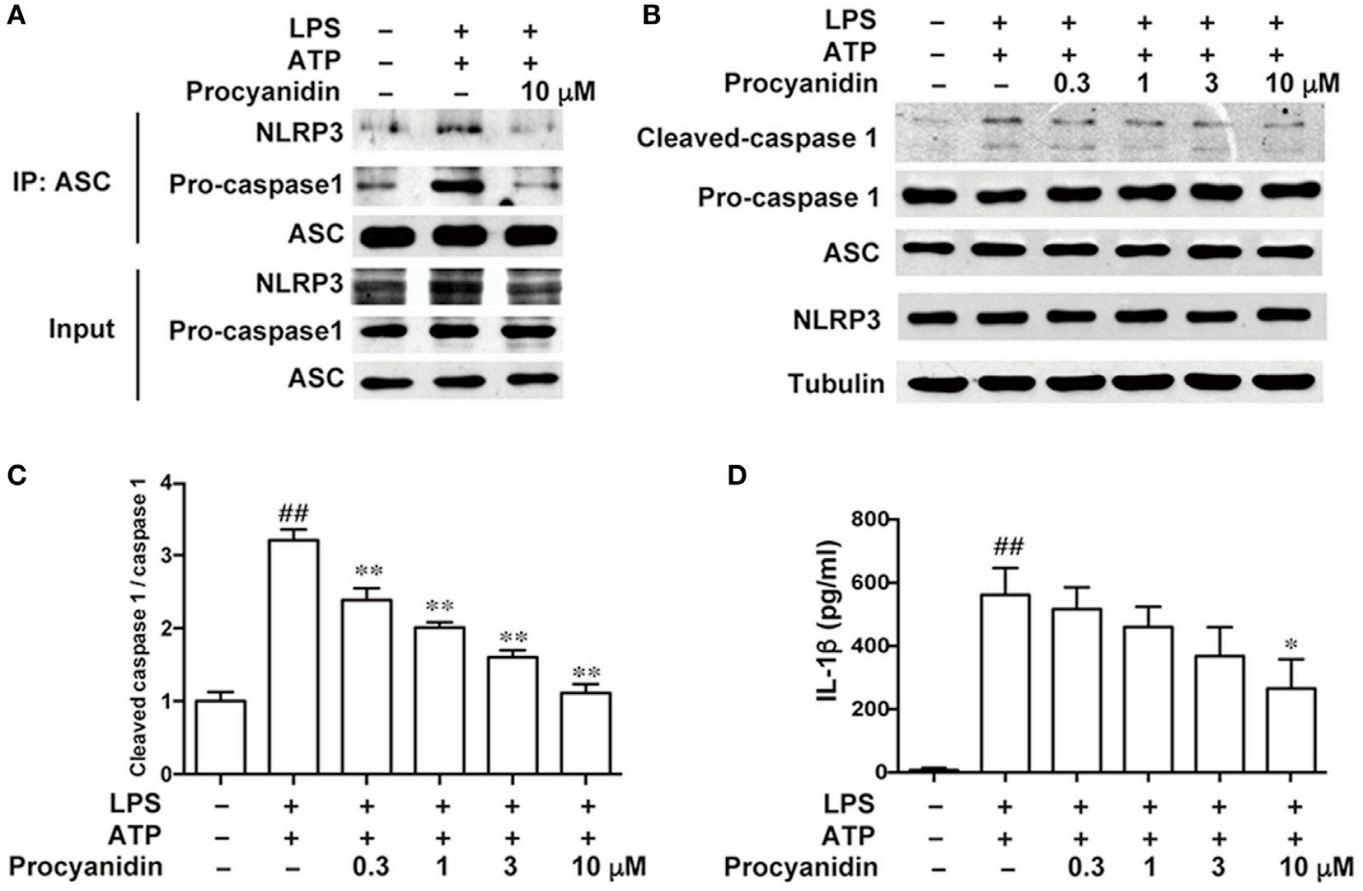
Procyanidin suppressed the activation of NLRP3 inflammasome in THP-1 cells. THP-1 cells were pretreated with 500 nM phorbolmyristate acetate for 3 h and cultured with 100 ng/ml lipopolysaccharide (LPS) in the absence or presence of procyanidin (10 µM) for 3 h, followed by a 1 h incubation with 5 mM adenosine triphosphate (ATP). **(A)** Proteins were isolated and immunoprecipitated with an antibody against ASC. **(B,C)** Protein levels of pro-caspase 1, cleaved caspase 1, ASC, and NLRP3 were determined by western blot. **(D)** Released interleukin (IL)-1β in the supernatant was analyzed by ELISA. ^##^*P* < 0.01 vs. vehicle group, *P < 0.05, **P < 0.01 vs. LPS + ATP-treated group.

The NLRP3 inflammasome is important in macrophage activation. Upon ATP stimulation, NLRP3 is co-localized with the apoptosis-associated speck-like protein containing a CARD (ASC), which has a caspase recruitment domain adaptor and pro-caspase 1, to form the inflammasome complex, resulting in the cleavage of pro-caspase 1 into its mature form. Therefore, the formation of the inflammasome complex is the core event in the activation of the NLRP3 inflammasome. In addition, IL-1β is an important proinflammatory cytokine in colitis and must be cleaved from its inactive form (pro-IL-1β) to the mature form by caspase 1. Here, we found that procyanidin (10 µM) significantly block the formation of the NLRP3 inflammasome induced by LPS and ATP (Figure 3A), resulting in decreased inflammasome activation and IL-1β release (Figures 3B–D). Together, these results suggested that procyanidin can interrupt the assembly of the NLRP3/ASC/caspase1 complex, thus inhibiting inflammasome activation and IL-1β release.

### Procyanidin Inhibited the NF-κB Signaling Pathway in THP-1 Cells

**Figure 4:**
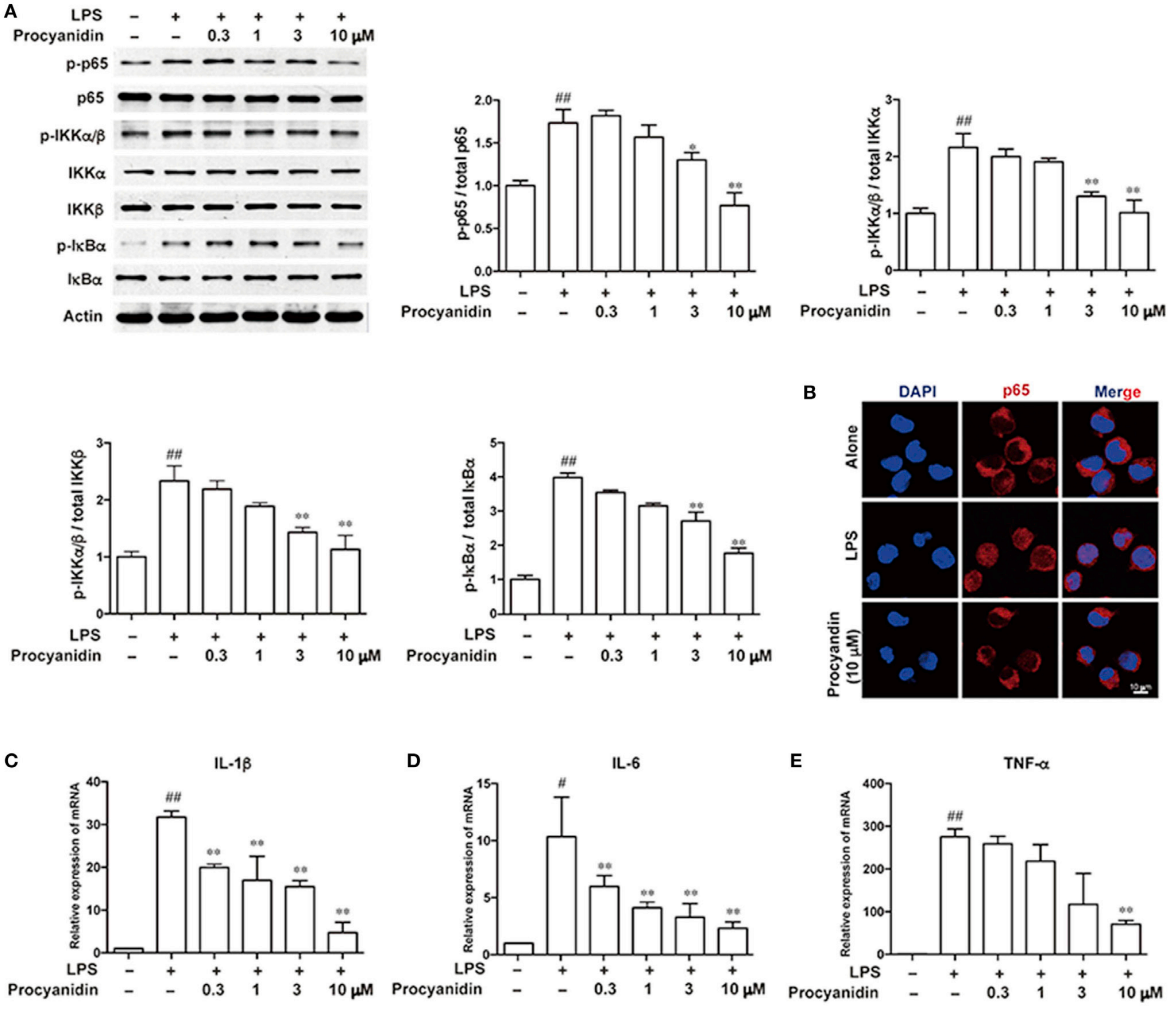
Procyanidin suppressed the NF-κB signaling pathway in THP-1 cells. THP-1 cells were treated with different concentrations of procyanidin (0.3, 1, 3, 10 µM) in the presence or absence of lipopolysaccharide (LPS) (100 ng/ml) for 30 min. **(A)** Protein levels of p-p65, p65, p-IKKα/β, IKKα, IKKβ, p-IκBα, and IκBα were determined by western blot. **(B)** Subcellular localization of p-p65 was examined by immunofluorescence by a confocal microscope. **(C–E)** mRNA levels of interleukin (IL)-1β, IL-6, and tumor necrosis factor-α were measured 6 h after LPS stimulation by real-time PCR. ^##^*P* < 0.01 vs. vehicle group, *P < 0.05, **P < 0.01 vs. LPS group.

Commonly, TLR4, which belongs to the family of pattern recognition receptors in macrophages, is responsible for immune responses upon activation of pattern-associated molecular patterns, such as LPS. Following LPS stimulation, ROS can be produced by TLR4 binding to NADPH oxidase 4 to produce superoxide (17). However, ROS scavengers can block the NF-κB activation stimulated by LPS (18). As shown in Figure 4A, in THP-1 cells, the NF-κB signaling pathway was significantly elevated following LPS (100 ng/ml) stimulation, as evidenced by higher levels of p-p65, p-IKKα/β, and p-IκBα, which can be inhibited by procyanidin (0.3, 1, 3, 10 µM) in a dose-dependent manner. In addition, under LPS stimulation (100 ng/ml) for 20 min, p65 translocated from the cytoplasm into the nucleus, which was significantly blocked by procyanidin (10 µM) (Figure 4B). In the nucleus, p65 promoted the transcription of pro-inflammatory cytokines, such as IL-1β, IL-6, and TNF-α. As shown in Figures 4C–E, procyanidin (0.3, 1, 3, 10 µM) markedly decreased the mRNA level after 6 h stimulation with LPS in THP-1 cells.

### Procyanidin Ameliorated DSS-Induced Experimental Colitis in Mice

**Figure 5:**
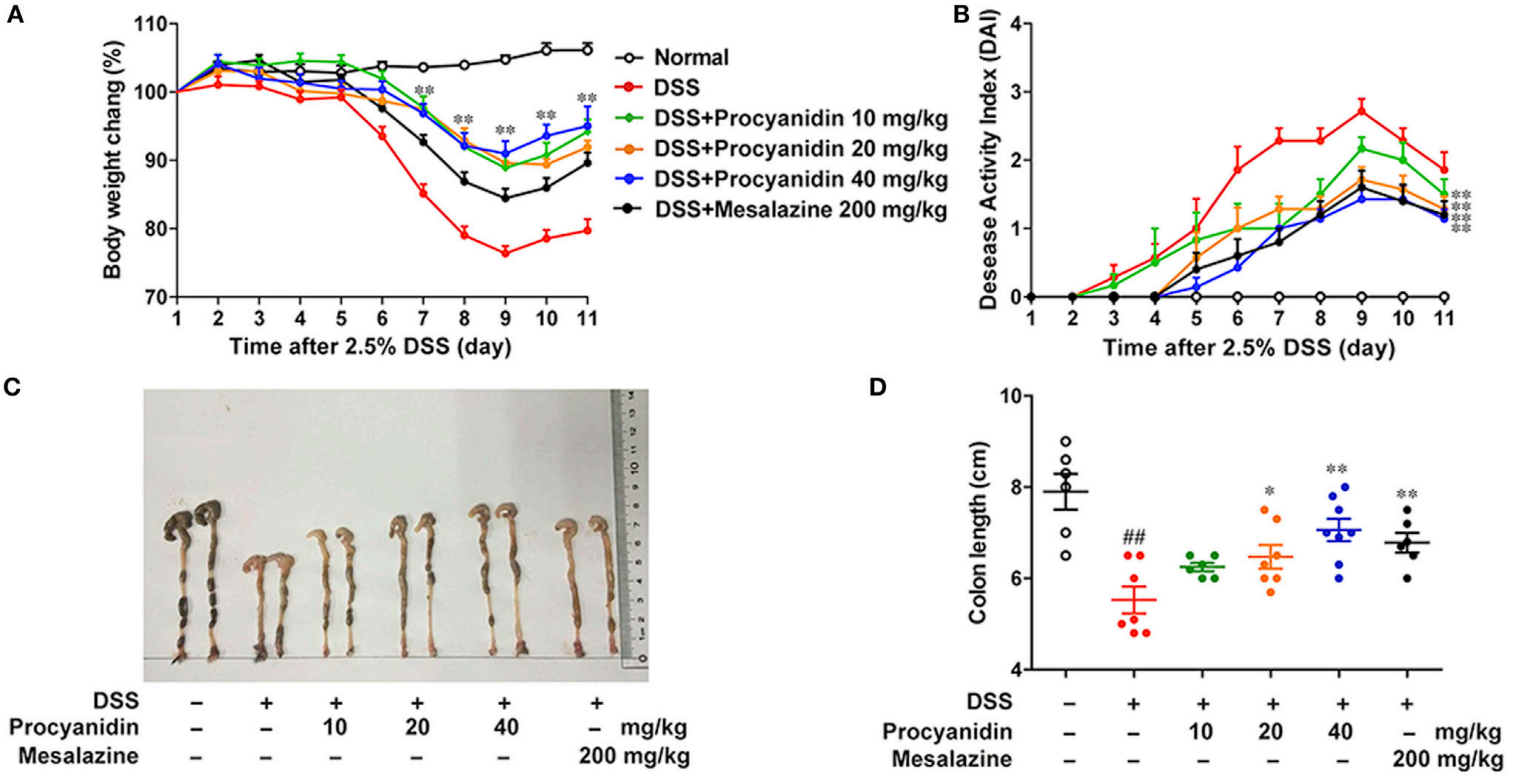
Procyanidin administration ameliorated Dextran sulfate sodium (DSS)-induced experimental colitis in mice. Mice were given 2.5% DSS in drinking water for nine consecutive days and then given normal drinking water for the next 2 days before being sacrificed. Procyanidin were orally administered daily (10, 20, and 40 mg/kg) from day 1 to day 11. **(A)** Body weight changes during the disease process. **(B)** Disease activity index was calculated. **(C,D)** Macroscopic images and length of the colon from each group were measured. Data are presented as the means ± SEM (*n* = 6–8 per group). ^##^*P* < 0.01 vs. normal, *P < 0.05, **P < 0.01 vs. DSS-treated group.

To determine the ability of procyanidin to modulate inflammation, we investigated the therapeutic effect of procyanidin on DSS-induced experimental colitis in mice. Mice were orally treated with 2.5% DSS for nine consecutive days and then normal water for 2 days, inducing a model of acute colitis that was characterized by severe weight loss, evident diarrhea, rectal bleeding, and shortened colon length. Mesalazine, a cyclooxygenase (COX) inhibitor, which reduces the risk of recurrence of IBD, is by far the most frequently used drug in IBD treatment (19), and in this study, we used mesalazine as a positive control in mice (20, 21). As shown in Figure 5A, compared to the vehicle-treated group, oral treatment of procyanidin, especially at a dose of 40 mg/kg, markedly attenuated weight reduction. In addition, procyanidin also significantly prevented disease progression, as shown by the DAI and colon length (Figures 5B–D). Furthermore, the effect of procyanidin at the highest dosage (40 mg/kg) was equivalent to that of mesalazine.

### Procyanidin Protected the Mouse Colon from the DSS-Induced Inflammatory Condition

**Figure 6:**
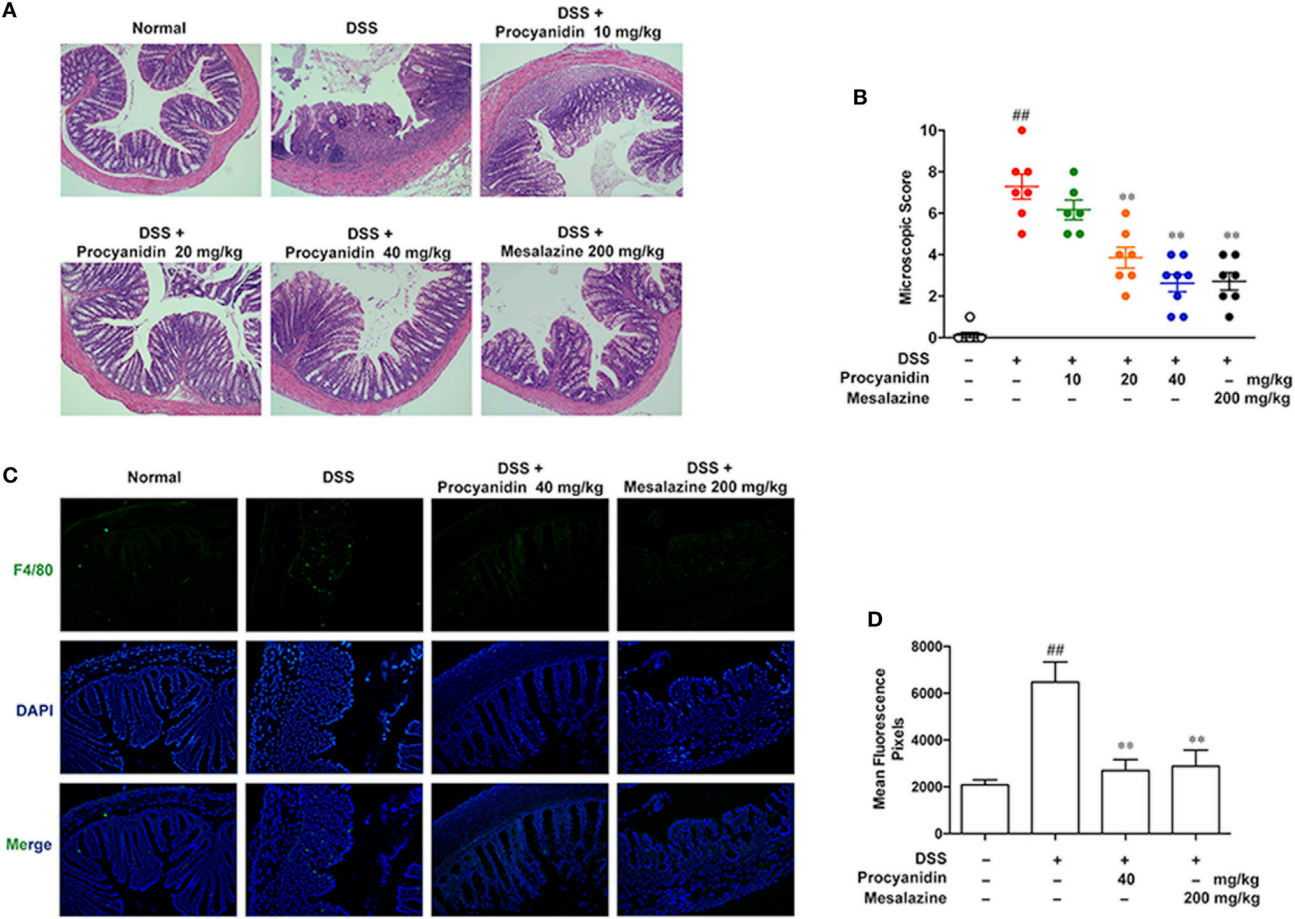
Procyanidin treatment prevented dextran sulfate sodium (DSS)-induced colon damage and macrophage infiltration in mice. **(A)** Serial sections of colon tissues were stained with H&E. **(B)** Histopathological scores of each group were determined. **(C)** Sections of colonic tissue were immunostained with antibody for F4/80-FITC (green) and DAPI (blue) and were observed using a confocal laser-scanning microscope. Magnification: 200×. **(D)** Fluorescence intensity of each group was determined. ^##^*P* < 0.01 vs. normal, **P < 0.01 vs. DSS-treated group.

Histology analysis implied that procyanidin exerted a better protective effect on DSS-induced inflammation in the colon (Figures 6A,B). Due to the critical role of macrophages in colitis, we next examined the effect of procyanidin on macrophage infiltration during colitis. As shown in Figures 6C,D, DSS induced obvious infiltration of F4/80 positive macrophages in the colon compared to vehicle-treated mice; by contrast, fewer infiltrating cells were observed in colonic samples from mice treated with procyanidin or mesalazine.

### Procyanidin Suppressed the Increased Expression of MMP9 *In Vivo*

**Figure 7:**
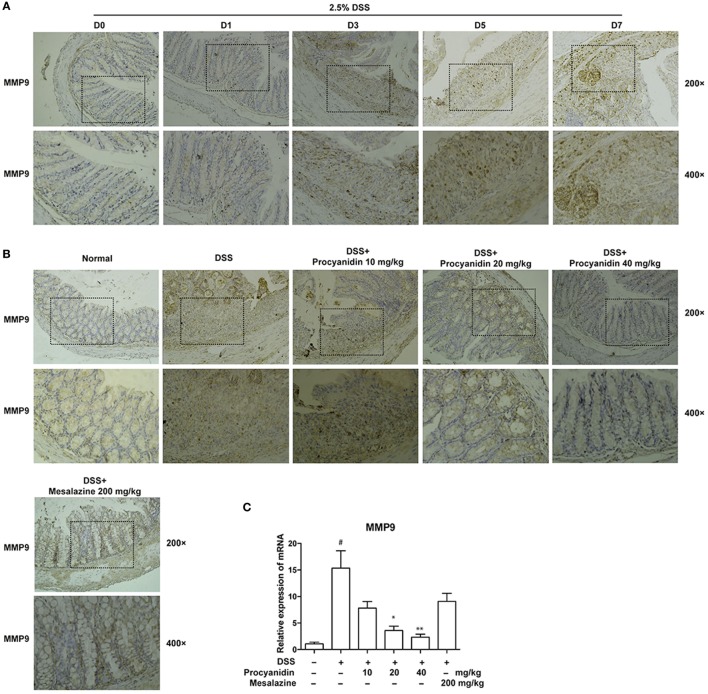
MMP9 expression was elevated after dextran sulfate sodium (DSS) administration and was inhibited by procyanidin treatment in mice. **(A)** Mice were orally administered 2.5% DSS for various times (0, 1, 3, 5, and 7 days). Paraffin-embedded colon tissue sections were stained for MMP9. **(B)** Paraffin-embedded colon tissue sections from vehicle and procyanidin-treated group were stained for MMP9. **(C)** The mRNA expression of MMP9 was determined by real-time PCR. ^##^*P* < 0.01 vs. normal, *P < 0.05, **P < 0.01 vs. DSS-treated group.

To clarify the *in vitro* results, mice were administered with 2.5% DSS for 0, 1, 3, 5, and 7 consecutive days, and the colons were collected. As shown in Figure 7A, MMP9 was significantly elevated with the development of DSS-induced colitis in colonic tissues. Therefore, MMP9 is markedly correlated with the severity of inflammation. However, the high expression of MMP9 was significantly decreased when mice were treated with procyanidin (10, 20, and 40 mg/kg), which was in line with the *in vitro* results (Figure 7B). In addition to protein level, procyanidin also decreased the elevated mRNA level of MMP9 in a dose-dependently way (10, 20, and 40 mg/kg); however, the effect of mesalazine on MMP9 expression was not significant (Figure 7C).

### Procyanidin Inhibited NLRP3 Inflammasome Activation in Mice

**Figure 8:**
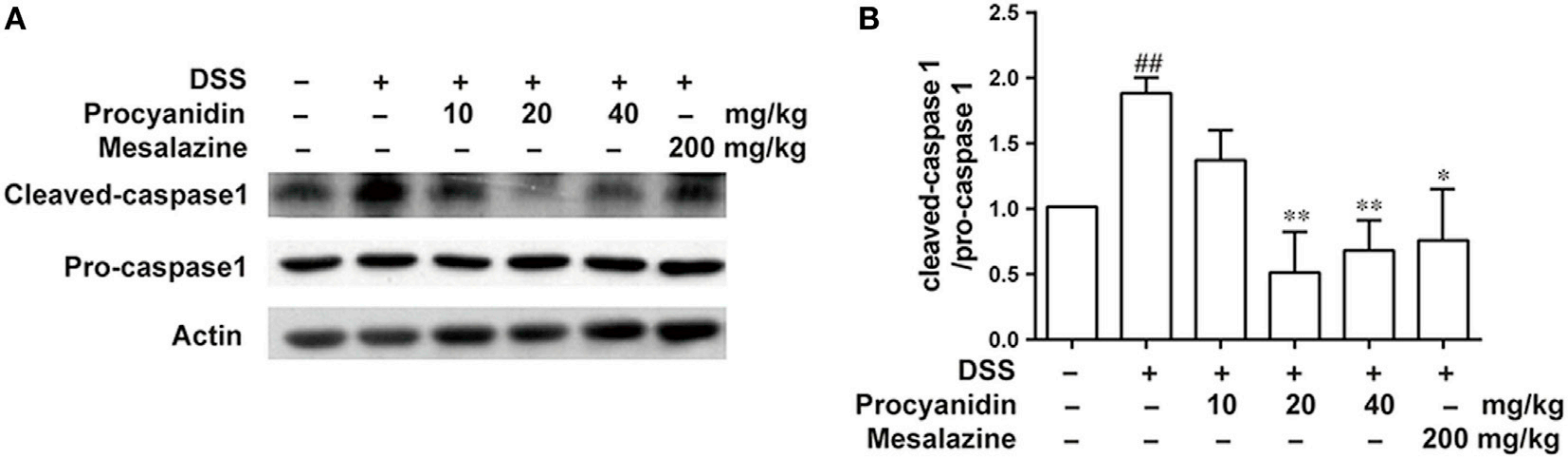
Procyanidin treatment inhibited dextran sulfate sodium (DSS)-induced activation of NLRP3 inflammasome in mice. **(A,B)** Colon tissue protein was extracted from mice, and the protein levels of pro-caspase 1 and cleaved caspase 1 were examined by western blot. ^##^*P* < 0.01 vs. normal, *P < 0.05, **P < 0.01 vs. DSS-treated group.

In accordance with the *in vitro* results, we also found that the NLRP3 inflammasome was markedly activated after DSS challenge in colonic tissue. However, procyanidin greatly inhibited the activation of the inflammasome, as evidenced by the decreased level of cleaved caspase-1 (Figures 8A,B).

### Procyanidin Suppressed the Activation of the NF-κB Signaling Pathway in Mice

**Figure 9:**
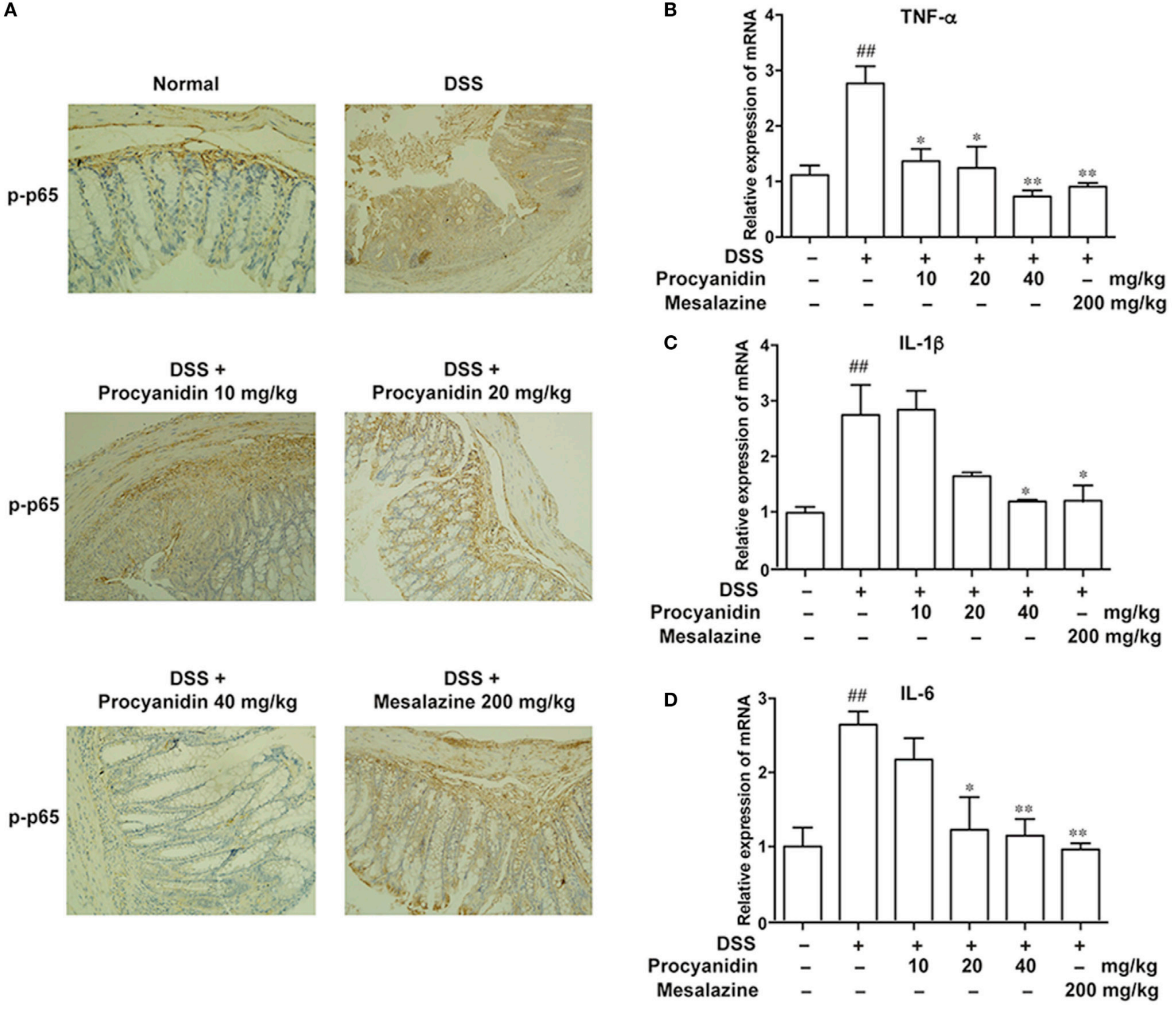
Procyanidin suppressed the activation of the NF-κB signaling pathway in mice. **(A)** Paraffin-embedded colon tissue sections from vehicle or procyanidin-treated group were stained for p-p65. **(B–D)** mRNA levels of interleukin (IL)-1β, tumor necrosis factor (TNF)-α, and IL-6 were determined by real-time PCR. ^##^*P* < 0.01 vs. normal, *P < 0.05, **P < 0.01 vs. dextran sulfate sodium (DSS)-treated group.

As the NF-κB signaling pathway plays a crucial role in inflammation mediated by macrophages, we assessed the effects of procyanidin on the activation of the NF-κB signaling pathway, which is characterized by elevated levels of p-p65. Immunohistochemical staining (Figure 9A) revealed that the phosphorylation level of p65 was markedly increased in the colon in the DSS group, while procyanidin significantly inhibited the p-p65 level. In addition, the mRNA levels of IL-1β, TNF-α, and IL-6 were also decreased after procyanidin treatment compared to DSS treatment alone (Figures 9B–D).

## Discussion

Inflammatory bowel disease is a chronic, refractory inflammatory disease that carries a high risk for colorectal cancer. The intestinal mucosa has the largest population of macrophages in the body and play vital roles in gut homeostasis (22). Macrophages have been demonstrated to be extensively involved in the pathogenesis of IBD. They can secrete many pro-inflammatory cytokines and chemokines, such as IL-1β, TNF-α, and IL-6, which in turn further trigger robust inflammatory reactions (23). Furthermore, it was observed that large number of activated macrophages can infiltrate in intestinal mucosa and regulation of macrophages-mediated immune responses can significantly attenuate experimental colitis in mice (13, 14). Oxidative stress, especially ROS, is a predominant factor in the mechanisms that underlie IBD (24). During the progression of colitis, excessive ROS are mainly produced by activated macrophages (24). ROS at high levels can induce many harmful responses, and serve as a common upstream of many inflammatory signaling, such as MMP9, NF-κB, and NLRP3 inflammasome pathway, which contribute much to the initiation and progression of colitis. Therefore, inhibiting the activation of macrophage and blocking ROS generation tends to be a protective method against colitis.

Our study aims to characterize the efficacy and mechanism of procyanidin, a strong ROS scavenger, in alleviating experimental colitis. Our *in vitro* data showed that procyanidin significantly reduced the generation of ROS and inhibited MMP9, NF-κB, and NLRP3 inflammasome signaling pathway in THP-1 cells (Figures 1–4). Therefore, we speculate that procyanidin can suppress the activation of macrophage *via* clearing ROS. In addition, the orally administration of procyanidin significantly attenuated colitis induced by DSS, and the effect of procyanidin, especially at the highest dosage (40 mg/kg), seems comparable to that of mesalazine (200 mg/kg) (Figure 5). Consistent with *in vitro* data, procyanidin significantly downregulated MMP9, NF-κB, and NLRP3 inflammasome signaling pathway in DSS-induced colitis in mice, which might due in part to the decreased number of macrophages (Figures 6C,D).

The matrix metalloproteinases (MMPs) is a family of zinc-dependent endopeptidases that degrade extracellular matrix proteins, and MMP9 is the predominant and most-studied member of this family (25). MMP9 has multiple functions, such as tumor growth and metastasis, and also plays a central role in inflammatory responses, such as tissue remodeling and wound healing. It has been suggested that in the pathogenesis of intestinal inflammation, MMP9 is consistently upregulated and serves as a biomarker for detecting disease activity in patients with IBD. The severity of DSS- or TNBS-induced colitis obviously decreased in MMP9^−/−^ mice (26, 27), and inhibition of MMP9 can also alleviate intestinal inflammation (28–30). The secreted MMP9 induced release of pro-inflammatory cytokines, affected the structural tissue integrity, and resulted in prolonged inflammation (31). In the present study, we found that MMP9 could be secreted by macrophages (Figure 2A), which accounted for most of the inflammatory response during colitis and we found, for the first time, that procyanidin could downregulate the high MMP9 expression induced by LPS stimulation in THP-1 cells (Figure 2B). In agreement with these *in vitro* data, increased MMP9 expression in both protein and mRNA level was also found in colonic tissue following DSS administration, and can be downregulated by procyanidin administration (Figure 7). Under pathological conditions, excessive ROS can directly or indirectly active MMPs and ROS can also decreased the level of tissue inhibitors of metalloproteinases, which balances MMPs activities *in vivo* (32). Therefore, we speculated that the inhibitory effect of procyanidin on MMP9 expression is based on its scavenger role for ROS (Figures 2B and 7B).

The key role of IL-1β is well-established in many immune cells, such as macrophages, dendritic cells, and neutrophils (33). Maturation of IL-1β from 31 to 17 kDa by NLRP3 inflammasome consisting of NLRP3, ASC, and caspase-1, is essential for this process (34, 35). Several models have described how the NLRP3 inflammasome is activated by ROS and that inhibition of the ROS levels can inhibit the activities of the NLRP3 inflammasome (13, 36–38). Under stimulation, ROS may serve as a triggering factor to activate NLRP3 to form macromolecular complexes with ASC and caspase1 (39). Formation and activation of the NLRP3 inflammasome further promotes the maturation of caspase1, which in turn cleaves pro-IL-1β into mature IL-1β (40). Although the role of NLRP3 in colitis is controversial, studies have demonstrated that NLRP3^−/−^ mice develop less severe colitis than wild-type mice and that targeting the NLRP3 inflammasome can significantly ameliorate experimental colitis (14, 41, 42). In the present study, we used LPS and ATP to activate the NLRP3 inflammasome in THP-1 cells. It was found for the first time that procyanidin greatly interrupted the formation of the NLRP3 inflammasome, resulting in downregulation of mature IL-1β release (Figure 3). In addition, we found that DSS induced the activation of NLRP3 in colonic tissue, but this was suppressed dose-dependently by oral procyanidin treatment (Figure 8), in accordance with the results *in vitro*.

It is widely acknowledged that ROS are essential for activating the NF-κB signaling pathway in response to LPS stimulation (43, 44). As shown in Figure 4, stimulation of LPS, which can bind to TLR4, activated the most important downstream molecule, NF-κB, in macrophages (45). The NF-κB family members include RelA (p65), NF-κB1 (p105 and p50), and NF-κB2 (p100 and p52) (46). RelB, c-Rel, and p65 are the most important members, which are obviously phosphorylated and translocated from the cytoplasm into the nucleus when the NF-κB signaling pathway is activated, resulting in the transcription of proinflammatory cytokines, such as IL-1β, TNF-α, and IL-6 (47). Here, we found that after LPS stimulation, the TLR-NF-κB signaling pathway was activated, and procyanidin can suppress the LPS-mediated phosphorylation and translocation of p-p65, resulting in decreased mRNA levels of IL-1β, TNF-α, and IL-6 (Figure 4). In addition, consistent with the results obtained from THP-1 macrophages, we observed the same effects of procyanidin in mice (Figure 9). It is worth noting that, although the expression of p-p65 in the mesalazine (200 mg/kg) group was higher than that of procyanidin (40 mg/kg), it was lower than the DSS group and was comparable to procyanidin at 20 mg/kg. The reduced mRNA levels of pro-inflammatory cytokines by mesalazine were equivalent to the procyanidin group, which revealed an overall attenuation of inflammatory conditions in mice. Furthermore, COX seems to be a downstream of NF-κB (48), so it might be reasonable that mesalazine, a COX inhibitor, ameliorates colitis without significant inhibition of p-p65 but decreased production of the pro-inflammatory cytokines.

Despite the potent inhibitory effect of procyanidin on MMP9, NF-κB, and NLRP3 inflammasome signaling in macrophages, we can suppose that procyanidin might also have effect on other cell types in colonic tissue, such as epithelial cells. As shown in Figures 6 and 9, MMP9 and p-p65 were widely distributed in colonic mucosal tissue. In fact, these signaling pathways also play important roles in intestinal epithelial cells during the initiation and progression of colitis. For example, there is a compelling evidence that epithelial MMP9 induces increased epithelial permeability by modulating cell–matrix interactions and wound healing (49). Epithelial NLRP3 was demonstrated a potent effect on epithelial integrity and intestinal homeostasis (50, 51). The NF-κB signaling pathway exacerbates inflammation and inhibition of NF-κB can regulate apoptosis of intestinal epithelial cells *in vitro* (52). However, we emphasized this study on the effect of procyanidin in macrophages for its pivotal role during the progression of colitis. For the other cells, further studies will be carried out.

Taken together, our data demonstrated that modulation of ROS by procyanidin provided a potential method for colitis treatment, downregulating the expression of MMP9, the activation of NF-κB signaling, and the NLRP3 inflammasome at the same time. Furthermore, procyanadin exerts an equivalent or even more impressive effect than mesalazine in some assays on colitis treatment. Our goal of future studies will be focused on the therapeutic effect of procyanidin in mice. More importantly, procyanidin can be administered orally; no side effects were observed after procyanidin administration at these dosage levels in mice, making the procyanidin treatment more attractive. Therefore, procyanidin might constitute an effective, safe, and convenient treatment option for patients experiencing severe diarrhea and abdominal pain with IBD.

## Ethics Statement

This study was carried out in accordance with the recommendation of Guide for the Care and Use of Laboratory Animals [Ministry of Science and Technology of China], and the Nanjing University Animal Care and Use Committee (NJU-ACUC). The protocol was approved by the Nanjing University Animal Care and Use Committee (NJU-ACUC).

## Author Contributions

QX, XW, and WL designed research. LC, QY, LH, JG, and QM performed research. LC and QY analyzed data. LC and XW wrote the manuscript.

## Conflict of Interest Statement

The authors declare that the research was conducted in the absence of any commercial or financial relationships that could be construed as a potential conflict of interest. The reviewer KL and handling Editor declared their shared affiliation.
